# Kenics Static Mixer Combined with Gas Sparging for the Improvement of Cross-Flow Microfiltration: Modeling and Optimization

**DOI:** 10.3390/membranes12070690

**Published:** 2022-07-04

**Authors:** Aleksandar Jokić, Nataša Lukić, Ivana Pajčin, Vanja Vlajkov, Selena Dmitrović, Jovana Grahovac

**Affiliations:** Faculty of Technology Novi Sad, University of Novi Sad, Bulevar cara Lazara 1, 21000 Novi Sad, Serbia; jokic@uns.ac.rs (A.J.); ivana.pajcin@uns.ac.rs (I.P.); vanja.vlajkov@uns.ac.rs (V.V.); selena.dmitrovic@uns.ac.rs (S.D.); johana@uns.ac.rs (J.G.)

**Keywords:** microfiltration, static mixer, gas sparging, response surface methodology, desirability function, permeate flux, specific energy consumption, microbial biopesticide, *Bacillus velezensis*

## Abstract

The use of membrane filtration as a downstream process for microbial biomass harvesting is hampered due to the low permeate flux values achieved during the microfiltration of fermentation broths. Several hydrodynamic methods for increasing permeate flux by creating turbulent flow patterns inside the membrane module are used to overcome this problem. The main goal of this study was to investigate the combined use of a Kenics static mixer and gas sparging during cross-flow microfiltration of *Bacillus velezensis* IP22 cultivation broth. Optimization of the microfiltration process was performed by using the response surface methodology. It was found that the combined use of a static mixer and gas sparging leads to a considerable increase in the permeate flux, up to the optimum steady-state permeate flux value of 183.42 L·m^−2^·h^−1^ and specific energy consumption of 0.844 kW·h·m^−3^. The optimum steady-state permeate flux is almost four times higher, whilst, at the same time, the specific energy consumption is almost three times lower compared to the optimum results achieved using gas sparging alone. The combination of Kenics static mixer and gas sparging during cross-flow microfiltration is a promising technique for the enhancement of steady-state permeate flux with simultaneously decreasing specific energy consumption.

## 1. Introduction

Biopesticides production depends on the method of cultivation broth downstream processing for sustainable and cost-effective use in agriculture [1]. Cultivation broth clarification in order to harvest microbial cells is the first step in downstream processing with the aim of concentrating the active component in the final biopesticide product. In biopesticides biotechnological production, this step is usually carried out by centrifugation or microfiltration [2].

One of the major drawbacks of microfiltration membrane processes that limit its extensive usage is mass transfer resistance through the membrane due to concentration polarization and membrane fouling. The most noticeable outcome of concentration polarization and membrane fouling is the decrease in permeate flux during cross-flow microfiltration due to the adsorption onto the membrane surface and inside the membrane pores. An essential step in restoring membrane permeability is membrane cleaning [3]. However, the capital and operating costs associated with frequent cleaning procedures, as well as the aggressiveness of cleaning, which causes damage to membrane surface, have steered researchers into developing techniques for permeate flux augment. Hence, a large number of research papers focus on the development of hydrodynamic methods for permeate flux enhancement by generating a turbulent flow pattern inside the membrane module, making it less susceptible to fouling [4,5,6,7,8,9]. 

Among hydrodynamic methods, turbulence promoters are by far the most commonly used method to increase permeate flux during cross-flow microfiltration. Turbulence promoters used to increase permeate flux can move within the membrane (dynamic) or be fixed relative to the membrane surface (static). Of the two types of turbulence promoters, static turbulence promoters are more investigated than dynamic turbulence promoters due to their numerous advantages: simpler installation, longer lifespan, lower investment and operational costs, and a wider range of operating feed flow rates and viscosities [10,11]. Static mixers increase permeate flux by splitting and redistributing the fluid flow inside the membrane, and, hence, by improving mixing in the radial and tangential direction, increasing the shear rate and inducing secondary flows near the membrane surface [12].

Besides static mixers, gas sparging is another technique that is employed as a means to successfully hinder membrane fouling and improve permeate flux [4,7,8,9,13,14,15,16,17,18,19,20,21]. By injecting gas into the feed, a two-phase (gas–liquid) flow inside the membrane module is created. As a result of forming bubbles inside the membrane module, local eddies are induced and shear rate at the membrane surface is increased [9]. The amount of induced shear rate mostly depends on gas and liquid phase linear speed, as well as bubble diameter and gas–liquid flow regime [22]. The presence of gas bubbles in the membrane channel causes efficient removal of loosely bound macromolecules and particles, while breaking of the cake layer could be caused by gas bubbles bursting or coalescence [6,7]. 

Combining the two methods for improving permeate flux can result in even greater enlargement by amplifying the fouling mitigation capabilities of both methods [9]. There have been very few studies on the combination of static mixer and gas sparging during membrane filtration [9,23,24]. Derradji et al. [23] investigated the ultrafiltration of a polymer solution (sodium alginate) combining two-phase gas–liquid flow with a Sulzer turbulence promoter placed before the membrane. The results indicated that the combination of Sulzer static mixer and gas sparging ensures a well-mixed two-phase flow which results in high permeate flux. Oil/water emulsion was another feed used in ultrafiltration with combined usage of static mixer and gas sparging [24]. Although in the case of oil/water emulsion, gas sparging does not seem to be very effective for flux enhancement, it was found that combining gas sparging and the use of the static mixer (Kenics) resulted in high permeate fluxes with low energy consumption. 

Armbruster et al. [9] investigated the effect of gas sparging, Kenics mixers, a combination of gas sparging with Kenics mixers and a novel concept, the aerating Kenics mixer (combining mixer and sparging in one single device). The combination of a Kenics mixer with gas sparging yielded the highest reduction in fouling for filtration of an aqueous solution of 30 mg·L^−1^ humic acid sodium salt. The authors concluded that the range of economical operation is considerably widened, whilst high flux operation is enabled by the application of static mixers and external air-sparging, or with aerating static mixers. 

Response Surface Methodology (RSM) is an efficient method to determine the connection between factors affecting a process and the output response variables of that process [25]. It provides a way of selecting a few points in the design space to efficiently represent the experimental space and, in that way, reduces the number of experimental runs for investigating the factor influences and interactions between them on the selected response. RSM was successfully applied in several subject areas connected to membrane processes, such as membrane synthesis optimization [26,27], optimization of chemical cleaning [28] and membrane performance [29,30,31,32].

The main objective of this study was to combine Kenics static mixer and gas sparging (SMGS) to improve permeate flux during cross-flow microfiltration of *Bacillus velezensis* IP22 cultivation broth. For assessing the optimal potential for flux improvement, response surface methodology was applied, i.e., desirability function approach. It was expected that optimum operational parameters could expand the main limitations of gas sparging, providing a viable answer for a high flux and low-energy broth microfiltration method.

## 2. Materials and Methods

### 2.1. Production of Bacillus velezensis Cultivation Broth

*Bacillus velezensis* IP22, the strain used as an active component of microbial biopesticide, was isolated from fresh cheese and previously identified using 16S rRNA gene sequencing [33]. A detailed description of the cultivation of *Bacillus velezensis* IP22 in the bioreactor is given in our previous work [4]. 

Microfiltration separation efficiency and *Bacillus velezensis* cells viability were evaluated by the standard plate count method and by measuring the absorbance (optical density) of the cultivation broth, retentate and permeate at a wavelength of 600 nm. The concentration of *Bacillus velezensis* (g/L) was calculated using biomass dry weight and the volume of cultivation broth, retentate or permeate sample (20 mL), which was centrifuged [4].

### 2.2. Microfiltration Experimental Setup

The experimental setup for *Bacillus velezensis* IP22 cultivation broth cross-flow microfiltration was described in detail in our previous publication [34]. All microfiltration experiments were performed at 25 °C and with recirculation of retentate and permeate to maintain a constant volume of the feed mixture. The ceramic membrane (Tami Deutschland, Hermsdorf, Germany) used in the experiments had the following characteristics: length 250 mm, inner diameter 6 mm, outer diameter 10 mm, pore size 200 nm and specific surface area 0.00433 m^2^. The Kenics static mixer used in the experiments had 30 mixing elements with a diameter of 5mm and a total length of 230 mm. It consists of a series of helical mixing elements made from thin, flat strips, twisted through 180˚ to form helices. Helices turned around their main axis by 90° against the next element [35]. The pressurized air was introduced into the feed flow channel through the three-way valve without the diffusor. The air flow rate was measured by the mass flow controller type EL-FLOW F-201AV, with an accuracy of ±0.5% (Bronkhorst, The Netherlands). During cross-flow microfiltration, the time (t) necessary for gathering 20 mL permeate (V) through the specific membrane surface area (A) was measured and the flux value (J, L·m^−2^·h^−1^) was calculated [4]:

(1) $$J=\frac{V}{A\cdot t}$$

Specific energy consumption (E, kW·h·m^−3^) is equal to the ratio of sum of hydraulic and pneumatic powers to the permeate flow rate and it was calculated according to Equation (2) [4]:

(2) $$E=\frac{Q_{L}\cdot \left(P_{S}-P_{D}\right)+\frac{\gamma }{\gamma -1}\cdot P_{D}\cdot Q_{G,D}\cdot \left[{\left(\frac{P_{S}}{P_{D}}\right)}^{\frac{\gamma -1}{\gamma }}-1\right]}{J\cdot A}$$

where Q_L_ (m^3^·h^−1^) is the feed flow rate, P_S_ (Pa) is pressure at the beginning of the membrane module, P_D_ (Pa) is pressure at the end of the membrane module, Q_G, D_ (m^3^·h^−1^) is the air flow rate at the pressure at the end of the membrane module (P_D_), γ is the specific heat ratio for air (1.4), J (m^3^·m^−2^·h^−1^)) is permeate flux and A (m^2^) is the specific membrane area.

### 2.3. Experimental Data Analysis—Modeling and Optimization

The effects of transmembrane pressure (TMP: 0.2–1 bar), superficial feed velocity (V_L_: 0.53–1.59 m·s^−^^1^) and superficial air velocity (V_G_: 0.0–0.46 m·s^−1^) were investigated by applying Box–Behnken’s experimental plan (Table 1). Selected responses were steady-state permeate flux and specific energy consumption. Experimental results were fitted using the second-degree polynomial models. Model adequacy was analyzed using the factorial ANOVA (analysis of variance). Coefficients and models were assessed by *p*-values, while the quality of the experimental data fitting was estimated using the Lack-of-fit, Pure error and R^2^ (coefficient of determination) values. All statistical analyses were performed at the significance level of 95% using the Statistica software (v. 13.5, Dell, Round Rock, TX, USA). The polynomial RSM models are usually used for optimization by the desirability function approach [26,27,35]. The Design-Expert software, v. 8.1 (Stat-Ease, Inc., Minneapolis, MN, USA) was used for generating the polynomial RSM models and optimization by the desirability function approach.

## 3. Results

The Box–Behnken design with three factors at three levels and three replications in the central point (Table 1) was used as the experimental design to evaluate the effect of microfiltration operational conditions (transmembrane pressure, TMP − *X*_1_, superficial feed velocity, V_L_ − *X*_2_, and superficial air velocity, V_G_ − *X*_3_) on steady-state permeate flux (J) and specific energy consumption (E). It included 15 experiments, and the experimental results were fitted with the second order polynomial model shown by Equation (3):

(3) $$Y=b_{0}+\overset{3}{\underset{i=1}{\sum }}b_{i}X_{i}+\overset{3}{\underset{i=1}{\sum }}b_{ii}X_{i}{}^{2}+\overset{2}{\underset{i=1}{\sum }}\overset{3}{\underset{j=1}{\sum }}b_{ij}X_{i}X_{j}$$

where *b*_0_ is the intercept term, *b_i_* are linear, *b_ii_* are quadratic and *b_ij_* are interaction effects, *X_i_* are input variables while *Y* is the response variable for either permeate flux or specific energy consumption. 

The results of fitting, including estimated values of linear (*b*_1_, *b*_2_, *b*_3_), quadratic (*b*_11_, *b*_22_, *b*_33_) and interaction (*b*_12_, *b*_13_, *b*_23_) model coefficients with their *p*-values, are listed in Table 2. The entries in bold font represent statistically significant coefficients at a level of 95% (*p*-values < 0.05). It is evident from the presented results that the linear effects of all input variables, the interaction effects of transmembrane pressure and superficial feed velocity, as well as the quadratic effects of all three input variables have statistically significant effects on steady-state permeate flux. In the case of a model for specific energy consumption, linear effect of superficial feed velocity, interaction between transmembrane pressure and superficial feed velocity, and all three quadratic effects are statistically significant.

The obtained models were tested by performing the analysis of variance (ANOVA). According to ANOVA (Table 3), the proposed regression models for permeate flux and specific energy consumption during microfiltration of *Bacillus velezensis* IP22 cultivation broth with the combination of Kenics static mixer and gas sparging are statistically significant, with *p*-values less than 0.05. Furthermore, high determination coefficients (R² > 0.98) for both models indicate their excellent ability to predict response variable behavior.

The ANOVA results given in Table 3 show that the coefficient of determination was 0.995 and 0.989 for second-degree polynomial models for permeate flux and specific energy consumption, respectively. The model for permeate flux could explain 99.5% of the variation in response, whilst for specific energy consumption, the value is 98.9%, which indicates the excellent fitness of the models. The predicted R^2^ of 0.931 is in reasonable agreement with the adjusted R^2^ of 0.987 for permeate flux, whilst in the case of specific energy consumption, the predicted R^2^ of 0.853 is also in good agreement with the adjusted R^2^ of 0.969. Adequate precision compares the range of the predicted values at the design points to the average prediction error; a ratio greater than 4 indicates adequate model discrimination. In this study, ratios of 32.163 and 19.955 for steady-state permeate flux and specific energy consumption, respectively, indicate an adequate signal.

Based on the ANOVA of the regression, both models are significant. In the case of steady-state permeate flux, a high F-value (116.33) and a low *p*-value (*p* < 0.0001) were observed, as well as a non-significant lack-of-fit (*p*-value of 0.153). For specific energy consumption, an F-value of 48.81 and *p*-value of 0.0002 imply that the model is significant, along with a non-significant lack-of-fit (*p*-value of 0.304). Furthermore, small values of pure error (8.667 for permeate flux and 0.055 for specific energy consumption) indicated that variation caused by measurement error is insignificant. 

Therefore, the selected regression models could be used to analyze trends of responses. Second-degree polynomial models could be successfully applied to describe the effects of transmembrane pressure, superficial feed velocity and superficial air velocity on steady-state permeate flux and specific energy consumption during SMGS cross-flow microfiltration. 

**Figure 1:**
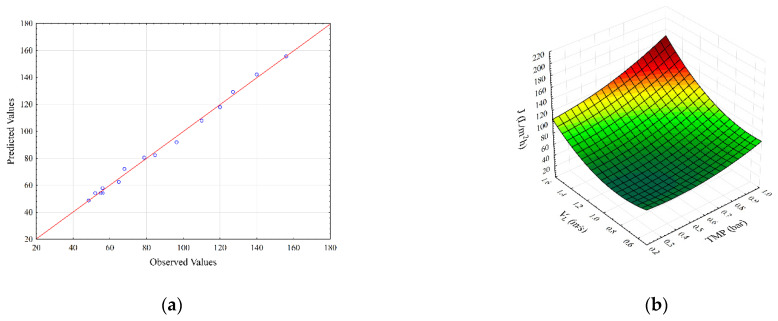

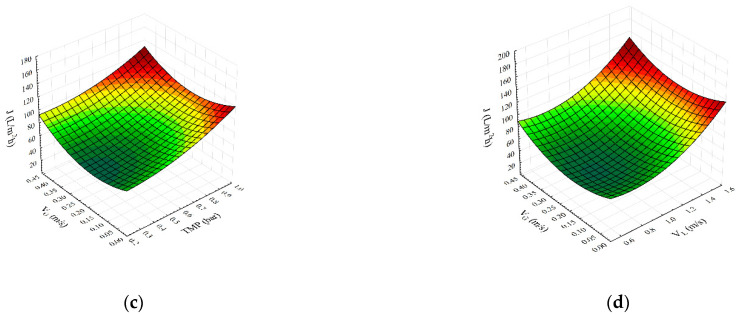
Parity plot (**a**) and response surface plots (**b**–**d**) representing the regression model for steady-state permeate flux during SMGS microfiltration of *Bacillus velezensis* IP22 cultivation broth.

**Figure 2:**
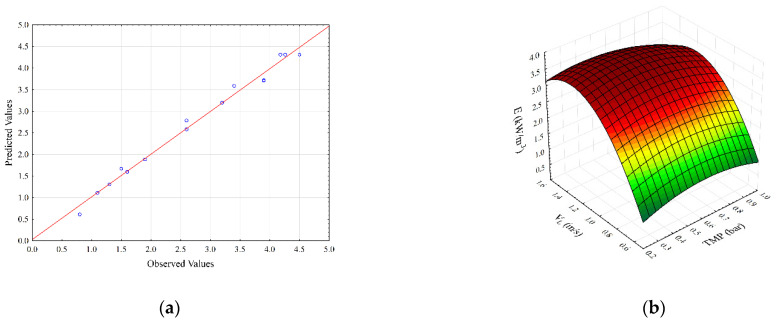

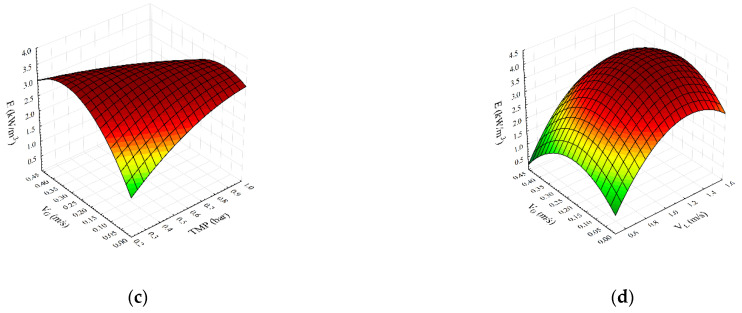
Parity plot (**a**) and response surface plots (**b**–**d**) representing the regression model for specific energy consumption during SMGS microfiltration of *Bacillus velezensis* IP22 cultivation broth.

Independent variables interactive effects on the steady-state permeate flux and specific energy consumption are illustrated by the 3D response surface plots (Figure 1 and Figure 2). 

The parity plot for steady-state permeate flux is shown in Figure 1a. As it can be seen, the second-degree polynomial model shows good agreement between experimental and predicted flux values. Figure 1b,c show the effects of transmembrane pressure (X1) with feed (X2) and gas (X3) velocities on the permeate flux, respectively. It can be seen (Figure 1b), that the maximum values of permeate flux are obtained for the highest transmembrane pressure and superficial feed velocity values. The steady-state flux increases with the increase in both feed velocity as well as TMP, indicating a strong synergistic effect between these variables (Figure 1b). On the other side, the increase in gas velocity resulted in lower flux improvements compared to the feed superficial velocity rise (Figure 1c). Transmembrane pressure in both cases had a positive effect on the flux enhancement. The influence of superficial feed and gas velocities on steady-state permeate flux is depicted in Figure 1d. The increase in both superficial velocities shows the positive effects on steady-state permeates flux.

The parity plot for specific energy consumption is shown in Figure 2a. As can be seen, the second-degree polynomial model shows good agreement between experimental and predicted flux values. Figure 2b,c show the effects of transmembrane pressure with feed and gas velocities on the specific energy consumption, respectively. It can be seen (Figure 2b) that maximum values of specific energy consumption are obtained for the highest superficial feed velocity values and lower transmembrane pressure values. The specific energy consumption increases with the increase in transmembrane pressure at lower superficial air velocities. On the other hand, at higher TMP values, specific energy consumption decreases at higher air velocities (Figure 2c). The influence of superficial feed and gas velocities on specific energy consumption is represented in Figure 2d. An increase in both superficial velocities results in a decline in specific energy consumption.

In this study, a desirability function method was applied for simultaneous optimization of investigated responses during SMGS microfiltration of *Bacillus velezensis* IP22 cultivation broth. Here, the maximum for steady-state permeate flux and the minimum for specific energy consumption were combined into one measurement called the desirability function. The selected responses were transformed to individual desirability values in a range from 0 to 1. The overall desirability of the process was computed as a geometric mean of the individual desirability functions [35]. The optimization results of the desirability function method are listed in Table 4. It can be seen that the maximum permeate flux and minimum specific energy consumption can be attained at the highest investigated factor levels: transmembrane pressure of 1 bar, superficial feed velocity of 1.59 m·s^−1^ and superficial gas velocity of 0.46 m·s^−1^. The high value of the overall desirability function (0.99) indicates that both optimization goals (maximum permeate flux and minimum specific energy consumption) were successfully accomplished. The predicted values of optimum permeate flux and specific energy consumption were 183.42 L·m^−2^·h^−1^ and 0.844 kW·h·m^−3^, respectively. On the other hand, in our previous study of microfiltration, assisted only by gas sparging [4], the optimum values for steady-state permeate flux of 48.57 L·m^−2^·h^−1^ and specific energy consumption of 2.37 kW·h·m^−3^ were predicted. By comparing the optimization results of SMGS microfiltration with the microfiltration, assisted only by gas sparging, it can be concluded that the presence of a static mixer considerably amounts to higher steady-state permeate flux and lower specific energy consumption.

Cell viability and concentration analyses have shown that *Bacillus velezensis* cells have been completely retained by the microfiltration membrane. Concentration of *Bacillus velezensis* biomass in the fresh cultivation broth was 0.37 g·L^−1^, while after the filtration experiments, biomass concentration in the retentate was 0.36 g·L^−1^. Spectrophotometric measurements (OD_600_) also confirmed this fact, and the absorbance values were 0.82 for the fresh cultivation broth and 0.78 for the retentate sample after microfiltration experiments. According to the results of the standard plate count method, *Bacillus velezensis* cells did not suffer either a significant change in the cell concentration or a decrease in cell viability due to increased shear stress caused by the mixer and air-sparging effects.

## 4. Discussion

Based on Figure 1b–d, the influence of operating conditions on steady-state permeate flux can be seen. With increasing transmembrane pressure, steady-state permeate flux increases for all values of superficial feed velocity (Figure 1b). With an increase in transmembrane pressure at lower values of superficial feed velocity, an increase in permeate flux is around 47%, whilst at higher values of superficial feed velocity, an increase in flux value is about 70%. The increases in steady-state permeate flux values were detected in the whole transmembrane pressure experimental range, contrary to the results obtained in the case of gas sparging without a static mixer [4]. In the case of gas sparging alone, the increase in transmembrane pressure lowers the steady-state flux due to the cell arrangement for smaller superficial feed velocity [4]. As reported in the literature, rod-shaped particles, such as *Bacillus velezensis*, tend to be oriented parallel to flow in the membrane channel so that they exhibit higher specific resistance and, consequently, lower flux values, even for the increased TMP [5,36,37,38]. When combining gas sparging with the static mixer, turbulence in the membrane channel is present, even for low feed velocities, so, in this way, orientation of rod-shaped particles, i.e., shear induced arrangement, is hindered from the start of the microfiltration process. In this way, flux is enhanced by reducing filtration cake resistance to permeate flow.

The effects of transmembrane pressure and superficial air velocity on the steady-state permeate flux are given in Figure 1c. At lower transmembrane pressure values, the increase in permeate flux is about 60%, while at higher values, the transmembrane pressure increase in permeate flux is only about 10%. The reason for this behavior can be found in the resistance of the filtration cake to the flow of smaller cultivation broth components. The combination of a Kenics mixer with gas sparging generated an increase in flux values in the whole range of superficial air values. Once again, the presence of a Kenics mixer had a decisive influence on flow conditions inside the membrane channel. The two-phase flow generated as a result of introducing air bubbles into the feed results in flux enhancement [4,6,7,8,39]. As reported in the literature, secondary induced flow patterns play a major role in the enhancement. It promotes local mixing influencing the flow in the membrane channel and thus reducing the resistance to mass transfer across the membrane [40]. In the case of gas sparging alone, a flux increase was observed up to a value of 0.25 m·s^−1^ for superficial air velocity [4], while in the case of experiments with the mixer and gas sparging combination, the flux increase was observed even for the velocities increased beyond this threshold. The cake structure becomes more compact (the particle packing becomes more regular) under the gas sparging [4,41], resulting in lower permeate flux, while the cake arrangement is avoided by the turbulence generated in the presence of a static mixer. At the highest superficial gas velocity, an enhancement in steady-state permeate flux of about 40% is observed, so more prominent improvement was achieved in comparison to only a 14% increase in a system with gas sparging alone [4].

The influence of superficial feed velocity and superficial gas velocity on steady-state permeate flux is depicted in Figure 1d. The insertion of the turbulence promoter into the membrane channel affects the flow of air bubbles within it, so the change in flow pattern causes significant differences in system performance. The steady-state flux values are significantly higher compared to gas sparging alone. The change in flow pattern through the membrane channel by inserting the Kenics static mixer results in smaller bubbles passing through the membrane channel closer to the membrane surface. Otherwise, smaller air bubbles would tend to stay grouped in the middle section of the membrane channel away from the membrane surface [42]. So, in this way, bubbles additionally contribute to the removal of the surface filtration cake along with the static mixer [9]. By increasing the values of superficial gas velocity up to 0.25 m·s^−1^, a plateau in permeate flux was reached in the case of gas sparging alone [4]. In the case of combining the effects of a static mixer and gas sparging, the plateau region was not observed. As the superficial air velocity rises, so larger bubbles are formed, creating slugs in the membrane channel. In the slug region of flow conditions, further increases in bubbles size, only increase the length of the slug which has no effect on wake size and does not contribute to the increased membrane surface scouring [42]. As a result, increasing superficial air velocity above 0.25 m·s^−1^ has little effect on permeate flux. Oppositely, when the static mixer is placed in the membrane channel, the flux rise is observed at all feed superficial velocities with increasing superficial air velocities (Figure 1d), suggesting that the Kenics mixer ensures a better mixing of the two-phase flow. Namely, the presence of a static mixer prevents the formation of larger air plugs. However, a higher increase in steady-state permeate flux (about 25%) was observed at lower superficial feed velocities, compared to the increase in steady-state permeate flux at higher values of superficial feed velocities (about 7%).

The influence of transmembrane pressure and superficial feed velocity on specific energy consumption is shown in Figure 2b. At lower superficial feed velocities, the effect of transmembrane pressure is insignificant. On the other hand, at the highest superficial feed velocity, by increasing the transmembrane pressure, a decrease in specific energy consumption occurs, due to the synergistic effect of both variables on the increase of permeate flux. As expected, an increase in superficial feed velocity leads to the sharp enhancement of specific energy consumption (up to 140%) for the whole range of investigated transmembrane pressure.

The influence of transmembrane pressure and superficial gas velocity on specific energy consumption is illustrated in Figure 2c. At lower superficial gas velocities, the values of specific energy consumption increased by about 100% by increasing the value of transmembrane pressure. Opposite to that, at higher superficial gas velocities, a further increase in transmembrane pressure decreases specific energy consumption. The lowest value of specific energy consumption of about 1.3 kW·h·m^−3^ is obtained at the highest transmembrane pressure and highest values of superficial gas velocity. The possible explanation for this behavior is in fact that the two-phase gas–liquid flow has greater influence on membrane surface cleaning. Compared to the microfiltration assisted only by gas sparging [4], in which the reduction in specific energy consumption under the operating conditions (high transmembrane pressure and superficial gas velocity) amounted to 52%, in microfiltration experiments with a combination of static mixer and gas sparging, a decrease in specific energy consumption is more significant and amounts to about 100%. 

The simultaneous influence of superficial feed and gas velocity on specific energy consumption is shown in Figure 2d. At lower superficial gas velocities, specific energy consumption increases. Given that a bubbly flow regime exists under these conditions, streamlined movement of bubbles due to the specific construction of Kenics static mixer does not contribute significantly to changes in the structure of the filtration cake layer, so the flux is not high enough to reduce energy consumption per m^3^. Therefore, specific energy consumption increases when lower permeate flux is achieved. However, in the slug gas–liquid flow regime, a different behavior is observed. Due to the effect of Kenics static mixer on slug flow, a significant increase in flux occurs, resulting in a decrease in specific energy consumption.

Optimization of operational conditions for SMGS microfiltration suggested that the optimum conditions are at the upper side of the experimental range of all variables. Contrary to the results of our previous study on microfiltration assisted only by gas sparging, significantly higher permeate flux values are accomplished with simultaneously lowering the specific energy consumption in the case of SMGS microfiltration. The optimum values of steady permeate flux are almost four times higher than in the case of applying gas sparging alone. On the other hand, high flux values result in a significant decrease in a specific energy consumption (per m^3^); in SMGS case almost three times lower, compared to the optimum specific energy consumption in gas sparging alone [4]. An additional experiment was conducted to validate the optimal solution for maximum permeate flux. The results of the verification experiment are in good agreement with the desirability function approach. The experimental values of optimum permeate flux and specific energy consumption were 178.53 L·m^−2^·h^−1^ and 0.882 kW·h·m^−3^, respectively.

## 5. Conclusions

The present study reports on the influence of combining Kenics static mixer and gas sparging on the performance of *Bacillus velezensis* IP22 cultivation broth cross-flow microfiltration. The study results confirm the achievement of the main goal, given that the combined use of a static mixer and gas sparging leads to a considerable increase in the permeate flux. Two-phase gas–liquid flow was completely altered in the presence of Kenics static mixer, resulting in a significant steady-state permeate flux improvement.

The optimization results for the maximum permeate flux and minimum specific energy consumption were attained at the highest investigated factor levels: transmembrane pressure of 1 bar, superficial feed velocity of 1.59 m·s^−1^ and superficial gas velocity of 0.46 m·s^−1^. The overall desirability function of 0.99 indicates that both optimization goals were accomplished. The predicted values of optimum steady-state permeate flux and specific energy consumption were 183.42 L·m^−2^·h^−1^ and 0.844 kW·h·m^−3^, respectively. The optimum steady-state permeate flux is almost four times higher, whilst at the same time, specific energy consumption is almost three times lower compared to the optimum results achieved using gas sparging alone.

## Figures and Tables

**Table 1:** Box–Behnken’s experimental plan for broth SMGS microfiltration experiments.

Experiment	Factors—Independent Variables			Responses—Dependent Variables	
	TMP (bar)	V_L_ (m·s^−1^)	V_G_ (m·s^−1^)	J (L·m^−2^·h^−1^)	E (kW·h·m^−3^)
1	0.2	0.53	0.23	48.5	1.6
2	1.0	0.53	0.23	68.0	1.5
3	0.2	1.59	0.23	96.3	3.9
4	1.0	1.59	0.23	156	3.2
5	0.2	1.06	0.0	56.0	1.9
6	1.0	1.06	0.0	110	3.9
7	0.2	1.06	0.46	78.7	3.4
8	1.0	1.06	0.46	120	1.3
9	0.6	0.53	0.0	64.9	1.1
10	0.6	1.59	0.0	127	2.6
11	0.6	0.53	0.46	84.6	0.8
12	0.6	1.59	0.46	140	2.6
13	0.6	1.06	0.23	55.0	4.3
14	0.6	1.06	0.23	52.0	4.5
15	0.6	1.06	0.23	56.0	4.2

TMP—transmembrane pressure, V_L_—superficial feed velocity, V_G_—superficial gas velocity, J—steady-state permeate flux, E—specific energy consumption

**Table 2:** Coefficients of regression models for steady-state permeate flux and specific energy consumption for broth SMGS microfiltration experiments.

Effects	Steady-State Permeate Flux (L·m^−2^·h^−1^)			Specific Energy Consumption (kW·h·m^−3^)		
	Coefficient		*p*-Value	Coefficient		*p*-Value
	Actual	Coded		Actual	Coded	
Intercept						
*b*_0_	**126.2**	**54.33**	**0.0002**	**−6.49**	**4.313**	**0.0002**
Linear						
*b*_1_	**−81.22**	**21.81**	**<0.0001**	6.46	−0.112	0.220
*b*_2_	**−157.3**	**31.66**	**<0.0001**	**11.87**	**0.912**	**<0.0001**
*b*_3_	**−141.2**	**8.18**	**0.0024**	15.98	−0.175	0.081
Quadratic						
*b*_11_	**77.86**	**12.46**	**0.0020**	**−2.85**	**−0.457**	**0.012**
*b*_22_	**90.45**	**25.41**	**<0.0001**	**−4.65**	**−1.307**	**0.0001**
*b*_33_	**460.9**	**24.38**	**<0.0001**	**−23.28**	**−1.232**	**0.0001**
Interaction						
*b*_12_	**47.41**	**10.05**	**0.0043**	−0.708	−0.15	0.244
*b*_13_	−34.51	−3.18	0.1797	**−11.14**	**−1.025**	**0.0003**
*b*_23_	−13.74	−1.68	0.4482	0.615	0.075	0.538

**Table 3:** ANOVA of regression models for steady-state permeate flux and specific energy consumption for broth SMGS microfiltration experiments.

Source	Response	DF	SS	MS	F-Value	*p*-Value
Model	J (L·m^−2^·h^−1^)	9	17364.6	1929.4	116.33	**<0.0001**
	E (kW·h·m^−3^)	9	22.662	2.518	48.805	**0.0002**
Residual	J (L·m^−2^·h^−1^)	5	82.93	16.58		
	E (kW·h·m^−3^)	5	0.258	0.052		
Lack-of-fit	J (L·m^−2^·h^−1^)	3	74.26	24.75	5.712	0.153
	E (kW·h·m^−3^)	3	0.202	0.068	2.434	0.304
Pure error	J (L·m^−2^·h^−1^)	2	8.667	4.333		
	E (kW·h·m^−3^)	2	0.055	0.028		
Total	J (L·m^−2^·h^−1^)	14	17447.5			
	E (kW·h·m^−3^)	14	22.920			
		**R^2^**	**Adj. R^2^**	**Pred. R^2^**	**Adeq. Precision**	
	J (L·m^−2^·h^−1^)	0.995	0.987	0.931	32.163	
	E (kW·h·m^−3^)	0.989	0.969	0.853	19.955	

J—steady-state permeate flux, E—specific energy consumption, DF—degree of freedom, SS—sum of squares, MS—mean squares, R^2^—coefficient of determination

**Table 4:** Optimization results obtained by the desirability function approach during SMGS microfiltration of *Bacillus velezensis* IP22 cultivation broth.

Factors—Independent Variables	Goal	Optimized Value
Transmembrane pressure, TMP (bar)	in range	1.0
Superficial feed velocity, V_L_ (m·s^−1^)	in range	1.59
Superficial air velocity, V_G_ (m·s^−1^)	in range	0.46
**Responses—Dependent Variables**	**Goal**	**Predicted Value**
Steady-state permeate flux, J (L·m^−2^·h^−1^))	maximize	183.42
Specific energy consumption, E (kW·h·m^−3^)	minimize	0.844
**Desirability function**	0.99	

## Data Availability

Not applicable.
